# Two year adverse outcomes of the magnetic levitated centrifugal continuous flow circulatory pump versus the axial continuous-flow pump for advanced heart failure: A systematic review and meta-analysis of randomized controlled trials

**DOI:** 10.1097/MD.0000000000019393

**Published:** 2020-02-28

**Authors:** Bing Tang, Hua Yang

**Affiliations:** Department of Cardiology, Jingzhou Central Hospital, The Second Clinical Medical College, Yangtze University, Jingzhou, 434020 Hubei, PR China.

**Keywords:** advanced heart failure, axial continuous flow pump, centrifugal continuous flow circulatory pump, pump replacement, pump thrombosis, right heart failure, sepsis, stroke

## Abstract

**Background:**

Due to advances in technology and medical devices, intra-thoracic left ventricular assisted devices such as the fully magnetically levitated centrifugal-flow pump may now prolong the life of patients with advanced heart failure. However, several concerns have been raised about pump thrombosis and durability of the device. We aimed to systematically compare the two year outcomes of magnetic levitated centrifugal continuous flow circulatory pump versus the axial continuous flow pump for advanced heart failure.

**Methods:**

Following the PRISMA guideline, online databases were searched for relevant trials based on centrifugal continuous flow circulatory pump and axial continuous flow pump in patients with advanced heart failure. The adverse clinical outcomes reported at 2 years follow-up were considered as the endpoints. This analysis was carried out by the RevMan 5.3 software whereby odds ratios (OR) and 95% confidence intervals (CI) were generated.

**Results:**

A total number of 1011 patients with advanced heart failure was included. At 2 years, pump thrombosis was not significantly different between the two groups, with OR: 0.43, 95% CI: 0.06–3.29; *P* = .42. However, pump replacement was significantly higher with the axial continuous-flow pump with OR: 0.36, 95% CI: 0.15–0.84; *P* = .02. Stroke, sepsis and bleeding events were not significantly different. In addition, outcomes such as right heart failure, cardiac arrhythmia, the need for right ventricular assisted device, respiratory failure, renal failure and hepatic dysfunction were also not significantly different.

**Conclusions:**

At a follow-up time period of 2 years, pump replacement was significantly higher with the axial continuous-flow pump in comparison to the magnetic levitated centrifugal continuous flow circulatory pump. However, no significant difference was observed with the other adverse outcomes.

## 1 Introduction

Heart Failure is becoming a critical concern in this aging population.^[1]^ Any acute or chronic diseased condition can lead to heart failure and death in advanced cases. Due to advances in technology and medical devices, intra-thoracic left ventricular assisted devices^[2]^ such as the fully magnetically levitated centrifugal-flow pump are expected to prolong the life of patients with advanced heart failure.. However, several concerns have been raised about pump thrombosis and durability of the device.

In 2016, the Multicenter Study of MagLev Technology in Patients Undergoing Mechanical Circulatory Support Therapy with HeartMate 3 (MOMENTUM 3) showed that the fully magnetically levitated centrifugal-flow pump device was not associated with pump thrombosis at 6 months follow-up, and pump malfunction was not a major issue.^[2]^ However, the device was seldom assessed on a long-term basis.

Since the MOMENTUM 3 Trial was continued up to two years, and other trials which were based on a 2 year follow-up time period were also recently published, we aimed to systematically compare the two year outcomes of centrifugal continuous flow circulatory pump versus the axial continuous flow pump for advanced heart failure through this meta-analysis.

## 2 Methods

### 2.1 Searched databases and searched strategies

This analysis was based on studies which were randomized controlled trials, and therefore, the Preferred Reporting Items for Systematic Reviews and Meta-analyzes (PRISMA) guideline was followed during the search of studies.^[3]^

Electronic/Online databases including the bibliographic database of life sciences and biomedical information: Medical Literature Analysis and Retrieval System Online (MEDLINE), the biomedical and pharmacological bibliographic database of published literature designed to support information managers and pharmacovigilance: Excerpta Medica dataBASE (EMBASE), Cochrane Central and http://www.ClinicalTrials.gov were carefully searched for relevant trials based on centrifugal continuous flow circulatory pump and axial continuous flow pump in patients with advanced heart failure by using the following searched terms:

(a) Centrifugal continuous flow circulatory pump versus axial continuous flow pump;
(b) Magnetic levitated centrifugal continuous flow circulatory pump versus axial continuous flow pump;
(c) Centrifugal continuous flow circulatory pump and advanced heart failure;
(d) Axial continuous flow pump and advanced heart failure;
(e) Centrifugal continuous flow circulatory pump;
(f) Axial continuous flow pump;
(g) Magnetic levitated centrifugal continuous flow circulatory pump;
(h) Magnetic levitated centrifugal continuous flow circulatory pump and advanced heart failure;
(i) Advanced heart failure and circulatory flow pump;
(j) Continuous flow left ventricular assisted devices;
(k) Left ventricular assisted device (LVAD) and advanced heart failure;
(l) Heart failure and assisted ventricular devices;
(m) LVAD and heart failure;
(n) LVAD and advanced heart failure.

Each of the above mentioned electronic databases [MEDLINE, EMBASE, Cochrane Central, http://www.ClinicalTrials.gov] was searched for relevant English publications using the respective above mentioned search terms.

### 2.2 Inclusion and exclusion criteria

Trials were included if:

(a) They compared centrifugal continuous flow circulatory pump versus axial continuous flow pump;
(b) They involved only patients with advanced heart failure;
(c) They reported adverse clinical outcomes;
(d) They had a follow-up time period of two years.

Trials were excluded if:

(a) They were review articles, meta-analyses, observational studies or case-control studies;
(b) They did not involve patients with advanced heart failure;
(c) They did not report adverse clinical outcomes;
(d) They had a follow-up time period of less than 2 years;
(e) They were duplicated trials.

### 2.3 Types of participants, outcomes and follow-up time periods

All the participants which were included in this analysis were patients with advanced heart failure.

The outcomes which were assessed included (Table 1):

(a) Pump thrombosis;
(b) Pump replacement;
(c) Any stroke;
(d) Ischemic stroke;
(e) Hemorrhagic stroke;
(f) Other neurological events;
(g) Any bleeding;
(h) Bleeding requiring re-operation;
(i) Bleeding requiring blood transfusion;
(j) Gastrointestinal bleeding;
(k) Sepsis;
(l) Left ventricular assisted device (LVAD) drive-line infection;
(m) Local infection not associated with LVAD;
(n) Right heart failure;
(o) Any cardiac arrhythmia;
(p) Need for right ventricular assisted device;
(q) Respiratory failure;
(r) Renal failure;
(s) Hepatic dysfunction.

**Table 1:**
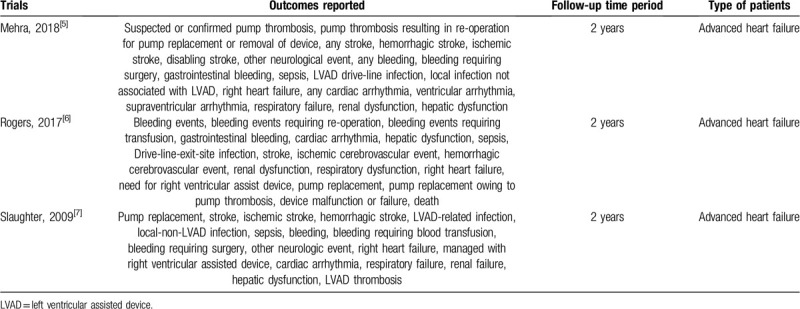
Types of participants, outcomes reported and follow-up time periods.

A follow-up time period of 2 years was considered relevant to this analysis.

### 2.4 Data extraction and quality assessment

Relevant data including the patients’ enrollment time period, the total number of participants assigned to the experimental and control groups, the baseline features of the participants, the total number of events reported, the outcomes which were assessed and the follow-up time periods were independently extracted by two reviewers (BT and HY).

Any disagreement which followed was resolved by consensus.

Quality assessment was carried out with reference to the criteria suggested by the Cochrane Collaboration.^[4]^

### 2.5 Statistical analysis

This analysis was carried out by the RevMan 5.3 software whereby odds ratios (OR) and 95% confidence intervals (CI) were generated.

Heterogeneity was assessed by:

- The Q statistic test whereby a *P* value less than .05 was considered as statistically significant;
- The I^2^ statistic test whereby the heterogeneity was increased with an increased I^2^ value; that is, the lower the I^2^ value, the lower the heterogeneity.

A fixed effects model (I^2^ < 50%) or a random effects model (I^2^ > 50%) was used based on the I^2^ value which was obtained.

In addition, sensitivity analysis was carried out by an exclusion method, whereby each study was excluded one by one and a new analysis was carried out each time to ensure that consistent results were obtained throughout.

Since this analysis included a small volume of study, publication bias was visually assessed through funnel plots which were generated by the RevMan software.

### 2.6 Ethical approval

This meta-analysis was based on previously conducted studies and did not contain any studies with human participants or animals performed by any of the authors.

## 3 Results

### 3.1 Searched outcomes

A total number of 265 publications were obtained. Following an initial assessment, 237 publications were eliminated since they were not related to the scope of this research. Twenty-eight (28) full-text articles were assessed for eligibility.

Further eliminations following assessment of the full-text articles were due to the following reasons:

- They were review articles (n = 2);
- They were not based on the comparison of centrifugal flow circulatory pump versus the axial continuous flow pump (n = 14);
- They did not report the adverse clinical outcomes (n = 2);
- They had a follow-up time period of less than 2 years (n = 1);
- They were duplicated trials (n = 6).

**Figure 1:**
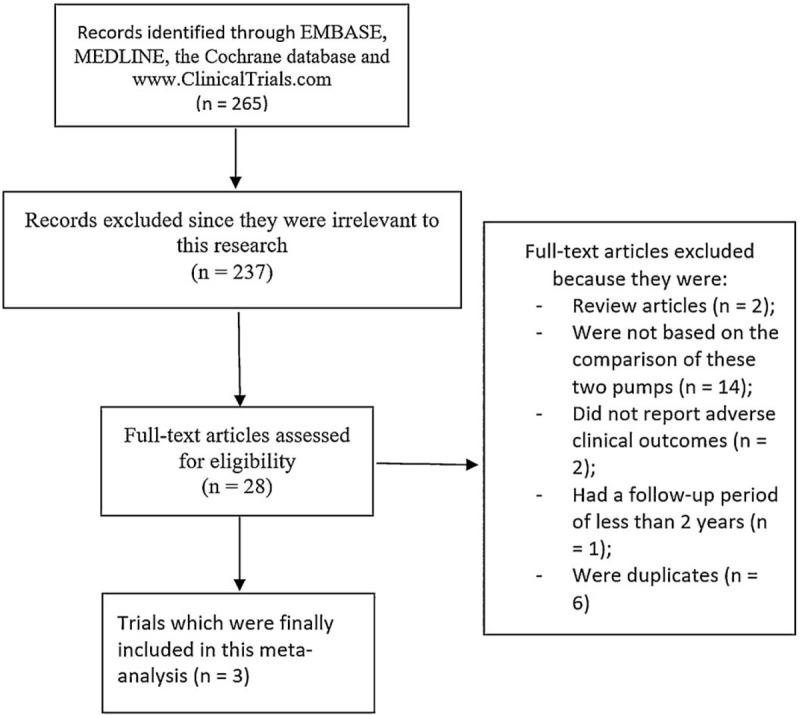
Flow diagram representing the study selection.

Finally, only three (3) trials^[5–7]^ were selected and confirmed for this meta-analysis as shown in Figure 1.

### 3.2 General and baseline features of the participants

A total number of 1011 patients with advanced heart failure was included in this analysis whereby 621 participants were assigned to the centrifugal continuous flow circulatory pump whereas 390 participants were assigned to the axial continuous flow pump as shown in Table 2. All the three studies were randomized controlled trials and the time period for patients’ enrollment was between years 2005 and 2015.

**Table 2:**
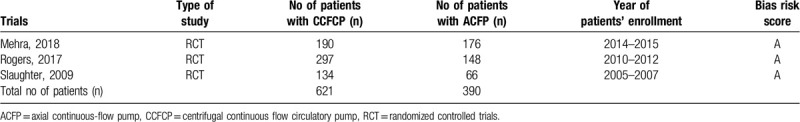
The General Features of the trials.

Following the bias risk assessment with reference to the Cochrane Collaboration, all the trials were allotted a grade A which implied a low risk of bias as shown in Table 2.

The baseline features of the participants were listed in Table 3. The patients had a mean age ranging from 59.0 to 66.2 years. Most of the participants were male patients with advanced heart failure having an average left ventricular ejection fraction ranging from 16.2 to 17.4%. The percentage of patients with ischemic cause of heart failure, with a history of stroke or atrial fibrillation, bridging for cardiac transplantation and their respective cardiac index which were reported in the original studies were listed in Table 3. According to Table 3, there was no significant difference in baseline features observed between patients who were assigned to either of the two groups.

**Table 3:**
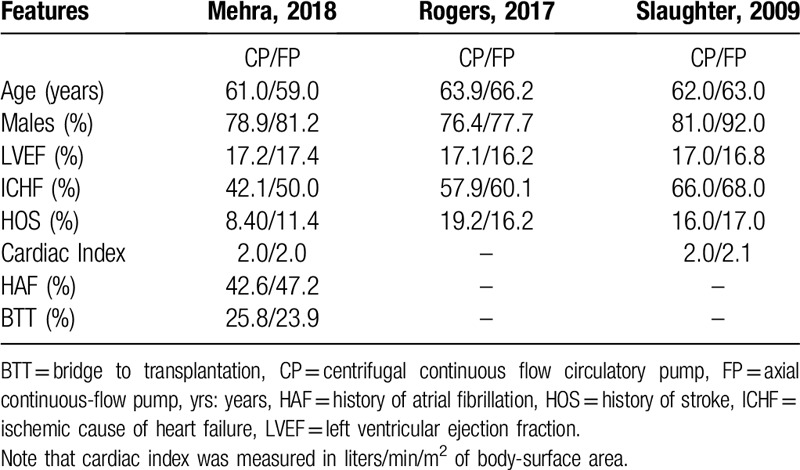
Baseline features of the participants.

The recommended anticoagulants which were used included: daily aspirin (81 mg to 100 mg) and warfarin monitored by an International Normalized Ratio (INR) between 2.0 and 3.0.

The frequency of regular follow-up was at 1 month, then 3 months, then 6 months after discharge, and then every 6 months until 2 years (Study Mehra2018).

### 3.3 Main results of this analysis

**Figure 2:**
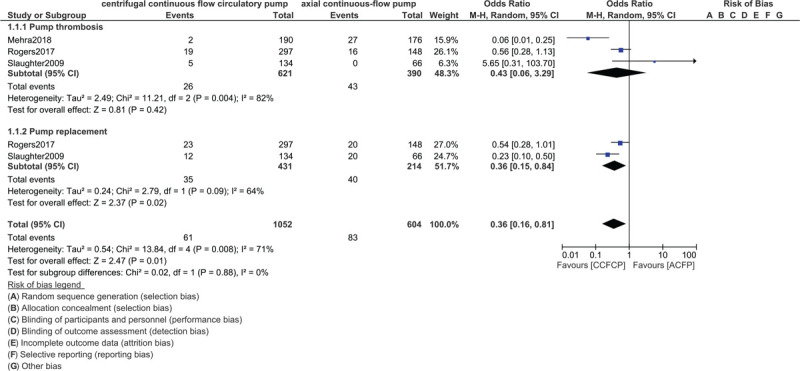
Comparing pump thrombosis and pump replacement between the centrifugal continuous flow circulatory pump versus the axial continuous flow pump.

At 2 years, when centrifugal continuous flow circulatory pump was compared with the axial continuous-flow pump in patients with advanced heart failure, pump thrombosis was not significantly different between the two groups, with OR: 0.43, 95% CI: 0.06–3.29; *P* = .42 (Fig. 2). However, pump replacement was significantly higher with the axial continuous-flow pump with OR: 0.36, 95% CI: 0.15–0.84; *P* = .02 as shown in Figure 2.

**Figure 3:**
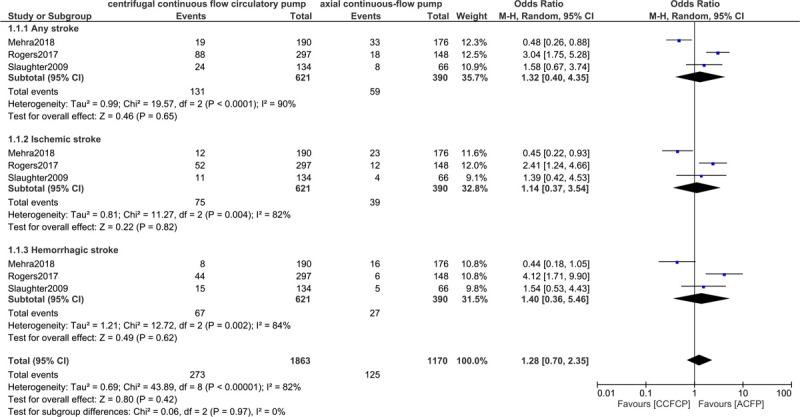
Comparing stroke between the centrifugal continuous flow circulatory pump versus the axial continuous flow pump.

**Figure 4:**
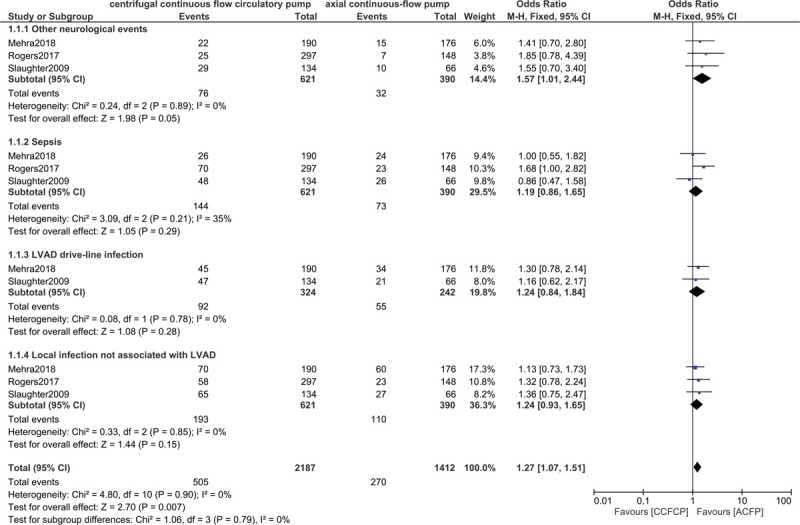
Comparing sepsis between the centrifugal continuous flow circulatory pump versus the axial continuous flow pump.

At 2 year follow-up, stroke including ‘any stroke’ (OR: 1.32, 95% CI: 0.40 – 4.35; *P* = .65), ischemic stroke (OR: 1.14, 95% CI: 0.37–3.54; *P* = .82) and hemorrhagic stroke (OR: 1.40, 95% CI: 0.36–5.46; *P* = .62) were not significantly different with either the centrifugal flow circulatory or the axial continuous-flow pump as shown in Figure 3. Other neurological events were also not significantly different (OR: 1.57, 95% CI: 1.01–2.44; *P* = .05) as shown in Figure 4.

In addition, sepsis, left ventricular assisted device (LVAD) drive-line infection, and local infection not associated with LVAD were also not significantly different at 2 years follow-up with OR: 1.19, 95% CI: 0.86–1.65; *P* = .29, OR: 1.24, 95% CI: 0.84–1.84; *P* = .28 and OR: 1.24, 95% CI: 0.93–1.65; *P* = .15 respectively as shown in Figure 4.

**Figure 5:**
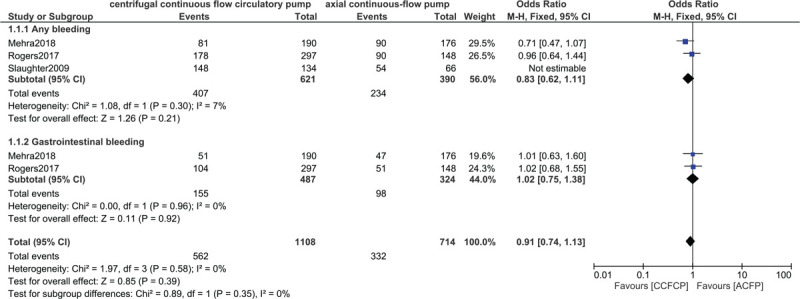
Comparing bleeding between the centrifugal continuous flow circulatory pump versus the axial continuous flow pump (part I).

**Figure 6:**
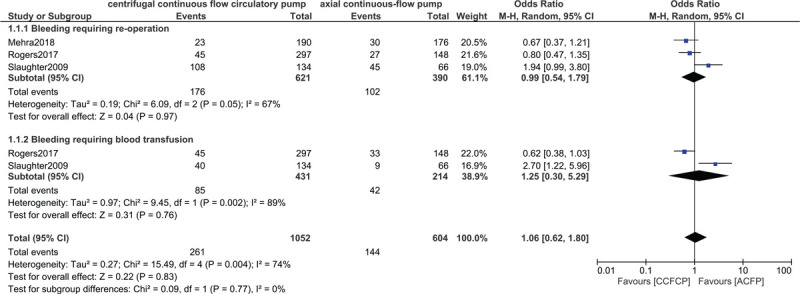
Comparing bleeding between the centrifugal continuous flow circulatory pump versus the axial continuous flow pump (part II).

Moreover, similar events representing ‘any bleeding’ and gastrointestinal bleeding were observed with OR: 0.83, 95% CI: 0.62–1.11; *P* = .21 and OR: 1.02, 95% CI: 0.75–1.38; *P* = .92 respectively as shown in Figure 5. Bleeding requiring re-operation and bleeding requiring blood transfusion were also not significantly different with OR: 0.99, 95% CI: 0.54–1.79; *P* = .97 and OR: 1.25, 95% CI: 0.30–5.29; *P* = .76 respectively as shown in Figure 6.

**Figure 7:**
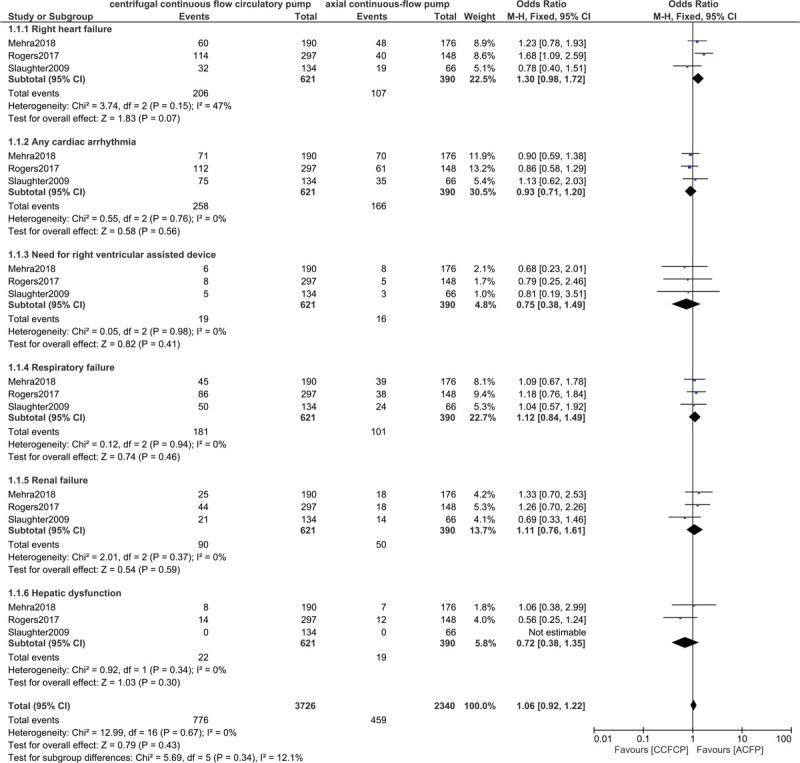
Comparing the other adverse clinical outcomes between the centrifugal continuous flow circulatory pump versus the axial continuous flow pump.

Right heart failure (OR: 1.30, 95% CI: 0.98–1.72; *P* = .07), Cardiac arrhythmia (OR: 0.93, 95% CI: 0.71–1.20; *P* = .56), the need for right ventricular assisted device (OR: 0.75, 95% CI: 0.38–1.49; *P* = .41), Respiratory failure (OR: 1.12, 95% CI: 0.84–1.49; *P* = .46), Renal failure (OR: 1.11, 95% CI: 0.76–1.61; *P* = .59) and Hepatic dysfunction (OR: 0.72, 95% CI: 0.38–1.35; *P* = .30) were not significantly different in these patients with advanced heart failure at 2 years follow-up as shown in Figure 7.

The results of this analysis have been listed in Table 4.

**Table 4:**
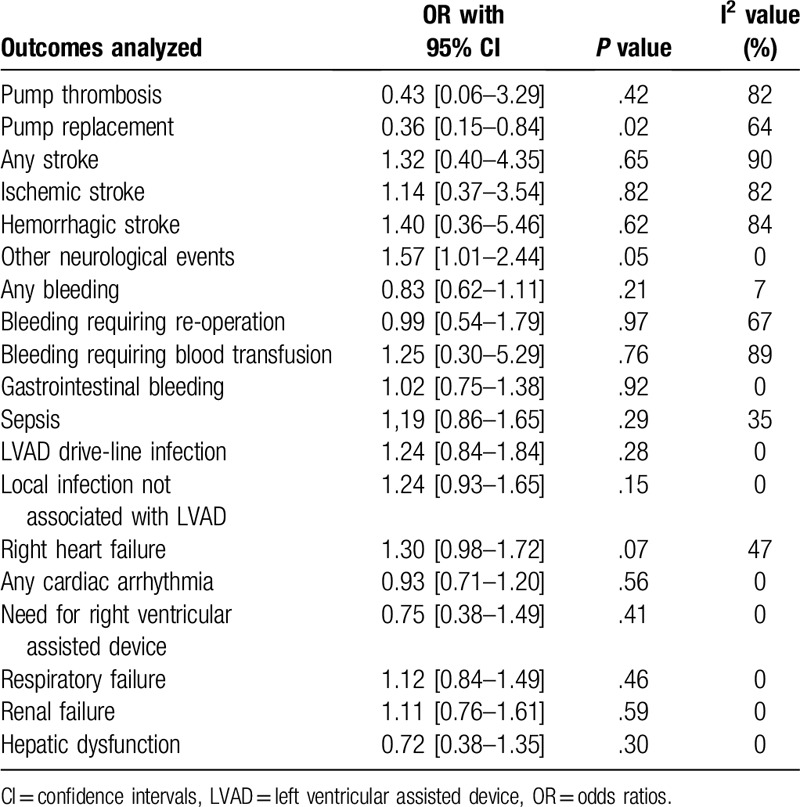
Results of this analysis.

**Figure 8:**
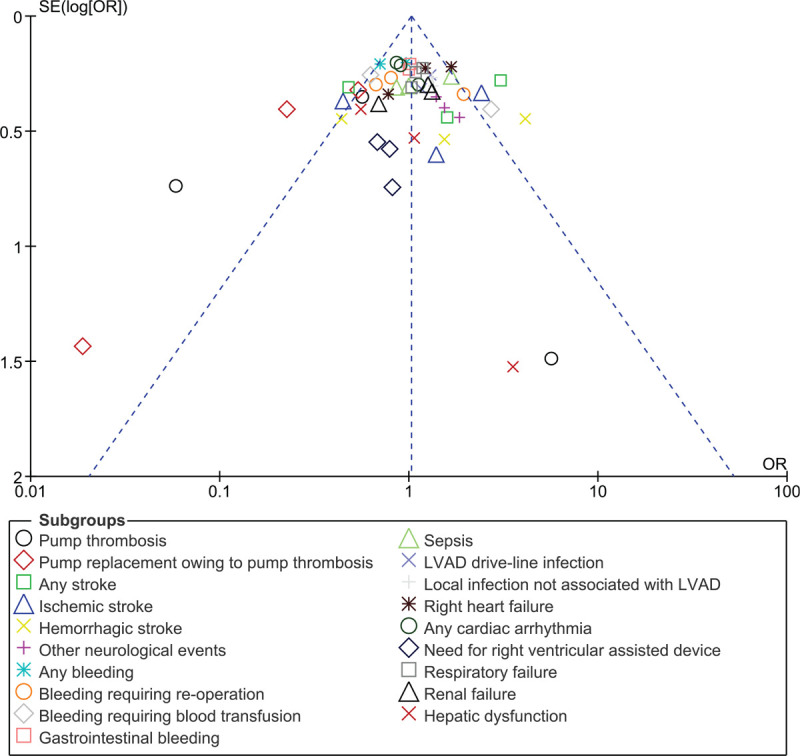
Funnel plot showing the assessment of publication bias.

Sensitivity analysis showed consistent results in all of the subgroups. Publication bias was visually assessed as shown in Figure 8.

## 4 Discussion

This current analysis showed that in patients with advanced heart failure, pump replacement was significantly higher with the axial continuous flow pump in comparison to the centrifugal continuous flow circulatory pump at 2 years. However, other outcomes including pump thrombosis, stroke, bleeding events, infections and different organ dysfunctions were not significantly different.

The possible reasons for pump replacement were pump thrombosis in a minority of patients, damage of the device lead, system related technical events such as communication fault leading to electrical failure, pump malfunction, sepsis including drive line infection, heart failure, persistent low pump flow due to obstructive outflow graft-twist or severe hemolysis.^[5–6]^ In study Slaughter et al^[7]^ of the 59 participants who were implanted with a pulsatile-flow LVAD, 21 pumps were replaced in 20 of the participants whereas among the 133 participants who were implanted with a continuous flow LVAD, 13 pump replacement were reported among 12 participants due to the breakage of the percutaneous lead, pump thrombosis and outflow elbow disconnection.

In this analysis, three randomized controlled trials were included. The Multicenter Study of MagLev Technology in Patients Undergoing Mechanical Circulatory Support Therapy with HeartMate 3 (MOMENTUM 3) having a follow-up time period of 2 years, is one among the trials which were used.^[2]^ However, the MOMENTUM 3 trial is a continuation of the previously published trial which had a follow-up period of only 6 months. Of the 294 participants with advanced heart failure which were included, 152 were assigned to the centrifugal continuous flow circulatory pump whereas 142 patients were assigned to the axial continuous flow pump. Similar to this current analysis, there was no significant difference in adverse outcomes, however, re-operation for pump malfunction or pump thrombosis was significantly higher with the axial continuous flow pump even at 6 months follow-up. Also, pump thrombosis occurred in 10.1% of the patients who were assigned to the axial continuous flow pump further supporting the fully magnetically levitated centrifugal-flow pump.

It would be interesting to know about the cost effectiveness of such devices. Recently, a Markov model was set up to assess the cost effectiveness of using these cardiac pump devices.^[8]^ All data were obtained from patients with advanced heart failure who were treated medically or with a continuous flow pump. Hospital claims were the source to determine the cost of such left ventricular assisted devices. When compared to patients who were managed medically, patients who were implanted with a continuous flow device had a higher 5 year cost ($ 360,407 compared to $ 62,856), quality adjusted life (1.87 vs 0.37 years) and life years. There was also a 75% reduction in incremental cost effectiveness ratio compared to that for the pulsatile flow device.

Nevertheless, thrombus formation has been a major concern for patients who were implanted with those left ventricular assisted devices. Even if the HeartMate II (axial) and the HeartWare HVAD (centrifugal) are being continually used in patients with advanced heart failure,^[9]^ it is high-time to focus more on the potential complications^[10]^ in other to further expand this technology for the treatment of such patients. The impact of anticoagulation on the reduction of stent thrombosis in these patients should also be considered.^[11]^

Moreover, even if our current study could not assess death outcome associated with these devices, the original studies showed a higher rate of death among those with a centrifugal flow device as compared to an axial flow device. To be more precise, in the ENDURANCE trial,^[6]^ a total number of 103 out of 297 patients who were implanted with the centrifugal flow device died compared to 39 death among 148 patients who were implanted with the axial flow device. Similarly, in another trial,^[7]^ 44% death occurred among those who were implanted with a continuous flow device compared to 27% death in the control group during a 2 year time period.

In comparison to previously published randomized controlled trials, this analysis consisted of a larger number of participants, giving an overview of the three trials. Also, all the trials had a similar follow-up period of 2 years, which might be another strength of this analysis, which would not be influenced by different follow-up time periods. At last, it should not be ignored that this idea is new in clinical medicine and in advanced progress of science and technology, and many research have yet to be carried out to better understand the clinical importance of these cardiac devices.

## 5 Limitations

Due to the limited number of participants in both groups, the results might have been affected. In addition, the total number of trials was limited in this analysis compared to other meta-analyses outside the scope of this topic. In addition, several subgroups showed a moderate to high level of heterogeneity, which might have affected the results. Other factors such as the duration of diseases, co-morbidities, and the use of different cardiac medications were not taken into consideration. A better analysis could also have been the comparison of adverse events from each individual pump and not classifying them by the type of flow.

## 6 Conclusions

At a follow-up time period of 2 years, pump replacement was significantly higher with the axial continuous-flow pump in comparison to the centrifugal continuous flow circulatory pump. However, no significant difference was observed with the other adverse outcomes. This hypothesis should be confirmed in future larger studies with even longer follow-up time periods.

## Author contributions

**Conceptualization:** Bing Tang, Hua Yang.

**Data curation:** Bing Tang, Hua Yang.

**Formal analysis:** Bing Tang, Hua Yang.

**Funding acquisition:** Bing Tang, Hua Yang.

**Investigation:** Bing Tang, Hua Yang.

**Methodology:** Bing Tang, Hua Yang.

**Project administration:** Bing Tang, Hua Yang.

**Resources:** Bing Tang, Hua Yang.

**Software:** Bing Tang, Hua Yang.

**Supervision:** Bing Tang, Hua Yang.

**Validation:** Bing Tang, Hua Yang.

**Visualization:** Bing Tang, Hua Yang.

**Writing – original draft:** Bing Tang, Hua Yang.

**Writing – review & editing:** Bing Tang, Hua Yang.
